# Rapid Metabolic Profiling of 1 μL Crude Cerebrospinal Fluid by Matrix-Assisted Laser Desorption/Ionization Mass Spectrometry Imaging Can Differentiate *De Novo* Parkinson’s Disease

**DOI:** 10.1021/acs.analchem.3c02900

**Published:** 2023-12-07

**Authors:** Theodosia Vallianatou, Anna Nilsson, Patrik Bjärterot, Reza Shariatgorji, Nuria Slijkhuis, Jordan T. Aerts, Erik T. Jansson, Per Svenningsson, Per E. Andrén

**Affiliations:** † Department of Pharmaceutical Biosciences, Spatial Mass Spectrometry, Science for Life Laboratory, Uppsala University, Uppsala SE-75124, Sweden; ‡ Department of Clinical Neuroscience, Karolinska Institute, Stockholm SE-17177, Sweden

## Abstract

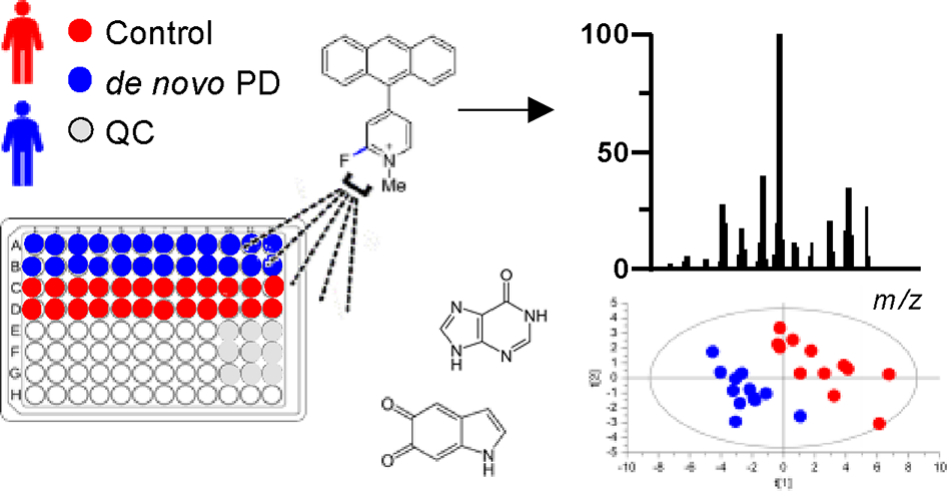

Parkinson’s disease (PD) is a highly prevalent neurodegenerative disorder affecting the motor system. However, the correct diagnosis of PD and atypical parkinsonism may be difficult with high clinical uncertainty. There is an urgent need to identify reliable biomarkers using high-throughput, molecular-specific methods to improve current diagnostics. Here, we present a matrix-assisted laser desorption/ionization mass spectrometry imaging method that requires minimal sample preparation and only 1 μL of crude cerebrospinal fluid (CSF). The method enables analysis of hundreds of samples in a single experiment while simultaneously detecting numerous metabolites with subppm mass accuracy. To test the method, we analyzed CSF samples from 12 *de novo* PD patients (that is, newly diagnosed and previously untreated) and 12 age-matched controls. Within the identified molecules, we found neurotransmitters and their metabolites such as γ-aminobutyric acid, 3-methoxytyramine, homovanillic acid, serotonin, histamine, amino acids, and metabolic intermediates. Limits of detection were estimated for multiple neurotransmitters with high linearity (*R*^2^ > 0.99) and sensitivity (as low as 16 pg/μL). Application of multivariate classification led to a highly significant (*P* < 0.001) model of PD prediction with a 100% classification rate, which was further thoroughly validated with a permutation test and univariate analysis. Molecules related to the neuromelanin pathway were found to be significantly increased in the PD group, indicated by their elevated relative intensities compared to the control group. Our method enables rapid detection of PD-related biomarkers in low sample volumes and could serve as a valuable tool in the development of robust PD diagnostics.

## Introduction

Parkinson’s disease (PD) is the second most common neurodegenerative disorder that progressively deteriorates movement control and execution. PD is considered one of the major causes of neurological disability, severely affecting life quality.^1^ Currently, diagnosis of the disease is mainly based on evaluation of clinical symptoms related to the motor performance of patients, whereas no chemical diagnostic tools have been implemented in clinical practice.^1−4^ However, the manifested motor symptoms can be associated with other neurodegenerative conditions, usually specified as atypical parkinsonism or PD Plus syndrome, complicating early and definite diagnosis of PD. Therefore, identification of specific and reliable biomarkers for the prognosis and early diagnosis of PD and their potential function as drug targets is of paramount importance.

Biomarker discovery via metabolomic methodologies is a rapidly evolving approach in precision medicine and the investigation of neurological disorders. Specific biomarkers have mainly been identified by analyzing plasma, serum, or urine samples owing to the simplicity of their collection.^5^ However, systemic changes do not always reflect central nervous system (CNS) pathologies because of the brain barriers, i.e., the blood-brain barrier and the blood-cerebrospinal fluid (CSF) barrier located in the choroid plexus.^6^ Therefore, the analysis of CSF samples may more accurately reflect the pathophysiological mechanisms underlying the neurodegenerative diseases, e.g., PD.

Liquid or gas chromatography coupled to mass spectrometry (LC–MS and GC–MS, respectively) or electrochemical detectors are the most frequently used techniques for CSF analysis. LC– and GC–MS-based metabolomics studies have shown a number of CSF metabolites significantly altered in PD.^7,8^ These include purine metabolites, such as 8-hydroxy guanosine, indicating DNA damage, tryptophan metabolites, creatinine, sphingolipids, and glycerophospholipids.^5,8−11^ Nevertheless, the limited ionization efficiency of monoamines, catecholamines, and indolamines, as well as other low abundant neurotransmitters, hampers their MS detection. In addition, tedious sample preparation and long LC–MS sequences can further reduce the applicability of the technique. An alternative technique for the detection of catecholamines in PD samples involves LC coupled to electrochemical detection.^12,13^ This approach is limited to a particular group of metabolites, mainly molecules susceptible to oxidative or reductive reactions.^14^

Both metabolomics and proteomics technologies have proven effective in investigating PD with the aim of identifying potential biomarkers for early detection, assessing disease progression, and evaluating treatment prognosis.^15−18^ Nevertheless, despite extensive efforts, the immediate and precise diagnosis of PD remains a challenge. Neither of these methodologies has been integrated into medical practice for PD diagnosis or for distinguishing between distinct stages of the disease. Significant progress has been made in the development of α-synuclein amplification assays (SAAs), which exhibit high specificity and sensitivity in distinguishing PD patients from controls.^19,20^ However, until now, the SAA remains a complex binary method that does not provide a quantitative measure of PD pathology.

We previously reported an MS imaging (MSI) method for the detection and visualization of comprehensive neurotransmitter systems and metabolites in brain tissue sections.^21−24^ We used a fluoromethylpyridinium-based reactive matrix to facilitate the covalent charge-tagging of molecules containing phenolic hydroxyl or primary or secondary amine groups, including dopaminergic and serotonergic neurotransmitters and their associated metabolites. The method was shown to improve the matrix-assisted laser desorption/ionization (MALDI)–MSI detection limit toward low-abundance neurotransmitters in brain tissue sections. We imaged multiple neurotransmitter pathways, i.e., GABAergic, dopaminergic, noradrenergic, serotonergic, and histaminergic pathways in rodent and nonhuman primate PD models.^23^ Here, we apply this method to CSF from 12 *de novo* PD patients, i.e., individuals not yet taking prescribed PD-specific medication^25^ and age-matched controls using ultrahigh mass resolution Fourier-transform ion cyclotron resonance (FTICR) mass spectrometry. The approach required minimum sample preparation and substantially reduced analysis time, i.e., numerous CSF samples could be analyzed within a few hours. In addition, we conducted sophisticated data analysis using in-house developed software to identify derivatized metabolites based on their mass-to-charge ratio (*m/z)*, with further validation achieved through MS/MS analyses. We also performed multivariate classification, i.e., partial least-squares discriminant analysis (PLS-DA), to identify significant metabolic differences between PD patients and control subjects. Multivariate analysis was found to be an appropriate tool,^26^ given the population size, to highlight a number of molecules significantly separating the two investigated groups. Thorough model validation and further investigation reduced this set to a smaller number of metabolites significantly affected by PD, primarily including neuromelanin-related species. Our method served as a highly effective screening tool even for a limited-size data set and could facilitate future cohort studies aimed at PD biomarker discovery.

## Results

### Overview of Data

CSF from 12 *de novo* PD patients (PD group) was compared to 12 age-matched controls (control group) using MALDI-MSI. The mean ages were 66.0 (±3.0) and 67.6 (±2.8) years for the PD and control groups, respectively. No significant age difference was observed between the two groups (Figure S1). An equal number of males and females was included in each group. All samples were analyzed in duplicate, and quality control (QC) samples were used to evaluate the experiment. The total MSI analysis time was 4 h, making the approach suitable for rapid CSF profiling.

The obtained MSI spectra were normalized by root-mean-square (RMS) normalization, which has been shown to give optimal performance in untargeted MSI experiments performed in FTICR instruments.^24,27^ Maximum intensity values were extracted for approximately 2900 peaks (*m*/*z* values) using SCiLS (Bruker Daltonics) software for data analysis and hypothesis testing. To limit irrelevant *m*/*z* intervals, filtering of the extracted features was performed (see the Methods section). Prior to further analysis, principal component analysis (PCA) was performed to obtain an initial overview of the data. After outlier detection and removal and further optimization (see the Methods section), a 10-component PCA (*R*^2^*X* = 0.738, *Q*^2^ = 0.55) was derived (Figure S1), based on which supervised data analysis was performed.

### Limit of Detection Determination

Calibration curves were constructed from the average intensities of isotope-labeled standards spiked into a control CSF sample. Overall, good linearities (*R*^2^ > 0.99) were obtained, and the limit of detection (LOD) of the measured standards was in the range of 16–380 pg/μL (Table S1). The corresponding endogenous neurotransmitters detected in the CSF were homovanillic acid (HVA), γ-aminobutyric acid (GABA), norephinehrine (NE), and serotonin (5-hydroxytryptamine, 5-HT). However, 5-HT was detected at a lower level than the LOD for 5-HT-*d*_4_. The calculated concentrations of the corresponding neurotransmitters were estimated from the obtained equations and were in the range of 0.1–0.23 ng/μL (Table S1). The neurotransmitters and corresponding metabolites were identified by mass matching (Table S2) and subsequent tandem MS (MS/MS). MS/MS was performed when the corresponding peak was of sufficient intensity to be isolated and fragmented.

### Metabolite Detection and Identification

An in-house automated method (see the Methods section) was used for the detection and identification of the CSF metabolome. The methodology exploited the high mass accuracy facilitated by FTICR-MS, as validated by measurements of isotope-labeled standards, resulting in mass errors consistently below ±1.4 ppm (as detailed in Table S2). To facilitate identification, the matching algorithm disqualified all candidates that could not be derivatized by FMP-10, meaning that molecules without a primary or secondary amine or phenolic hydroxyl group were removed. The automated identification algorithm detected approximately 320 metabolites available in the human metabolome database based on mass accuracy (Data S1). MS/MS spectra were acquired for a number of metabolites for which standards could be compared to obtain higher validation confidence.^28^ In parallel, MSI data were acquired for synthetic standards of approximately 70 different neurotransmitters and metabolites (Table S3) to compare with and refine the list of the computationally identified molecules. Subsequently, MS/MS spectra from selected standards were compared to the corresponding MS/MS spectra of CSF (Figures S2, S3, and S6 and Data S2). This process enabled the verification of the identity of 22 metabolites with high confidence (Table 1).

**Table 1:** List of Identified Amino Acids, Neurotransmitters, and Metabolites in the CSF Samples

metabolite	derivatization species	theoretical *m*/*z* ratio	experimental *m*/*z* ratio	mass error (ppm)	adjusted experimental *m*/*z* ratio	adjusted error (ppm)	structural validation	statistically significant
glycine	[M + FMP-10]^+^	343.14410	343.1448	1.95	343.1444	0.96	MS/MS	no
putrescine	[M + 2FMP-10]^+^	623.31692	623.3171	0.24	623.3170	0.14	MS/MS	no
alanine	[M + FMP-10]^+^	357.15975	357.1604	1.79	357.1600	0.81	MS/MS	no
GABA	[M+FMP-10-H_2_O]^+^	353.16484	353.1654	1.70	353.1651	0.71	MS/MS	no
serine	[M + FMP-10]^+^	373.15467	373.1553	1.66	373.1549	0.70	MS/MS	no
histamine	[M + 2FMP-10]^+^	646.29652	646.2966	0.08	646.2966	0.12	MS/MS	no
creatinine	[M + FMP-10]^+^	381.17099	381.1715	1.29	381.1711	0.34	MS/MS	no
valine	[M + FMP-10]^+^	385.19106	385.1917	1.69	385.1913	0.73	MS/MS	no
taurine	[M + FMP-10]^+^	393.12674	393.1272	1.14	393.1268	0.20	MS/MS	no
creatine	[M + FMP-10]^+^	399.18155	399.1822	1.58	399.1818	0.65	MS/MS	no
isoleucine/leucine	[M + FMP-10]^+^	399.20671	399.2072	1.28	399.2069	0.35	MS/MS	no
hypoxanthine	[M + 2FMP-10]^+^	657.23974	657.2395	–0.37	657.2396	–0.24	MS/MS	yes
tyramine	[M + 2FMP-10]^+^	672.30094	672.3007	–0.43	672.3008	–0.21	MS/MS	no
glutamine	[M + FMP-10]^+^	414.18122	414.1818	1.38	414.1814	0.46	MS/MS	no
methionine	[M + FMP-10]^+^	417.16313	417.1640	1.99	417.1636	1.08	mass accuracy^a^	no
DOPAL	[M + FMP-10]^+^	420.15942	420.1600	1.40	420.1596	0.50	mass accuracy^b^	no
acetyl-histamine	[M + FMP-10]^+^	421.20229	421.2031	1.95	421.2027	1.04	MS/MS	no
histidine	[M + FMP-10]^+^	423.18155	423.1821	1.28	423.1817	0.38	MS/MS	no
3-MT	[M + FMP-10]^+^	435.20671	435.2073	1.45	435.2070	0.57	mass accuracy^a^	no
DOPAC	[M + FMP-10]^+^	436.15434	436.1551	1.74	436.1547	0.87	mass accuracy^a^	no
arginine	[M + FMP-10]^+^	442.22375	442.2245	1.79	442.2242	0.95	MS/MS	no
DOPEG	[M + FMP-10]^+^	438.16999	438.1705	1.16	438.1701	0.30	mass accuracy^a^	no
5-HT	[M + FMP-10]^+^	444.20704	444.2077	1.42	444.2073	0.59	mass accuracy^a^	no
tyrosine	[M + FMP-10]^+^	449.18597	449.1865	1.16	449.1861	0.33	MS/MS	no
HVA	[M + FMP-10]^+^	450.16999	450.1706	1.44	450.1703	0.62	MS/MS	no
5-HIAA	[M + FMP-10]^+^	459.17032	459.1709	1.33	459.1706	0.52	MS/MS	no
tryptophan	[M + FMP-10]^+^	472.20195	472.2022	0.42	472.2018	–0.34	mass accuracy^a^	no
3-OMD	[M + FMP-10]^+^	479.19653	479.1973	1.67	479.1970	0.92	MS/MS	no
α-tocopherol	[M + FMP-10]^+^	698.49316	698.4932	0.03	698.4935	0.44	MS/MS	no
aminochromes^c^	[M + FMP-10]^+^	417.15975	417.1602	1.15	417.1600	0.60	MS/MS	yes
	[M + FMP-10]^+^	433.15467	433.1551	1.09	433.1549	0.55	MS/MS	yes

a MS/MS was performed on the standard compound. However, the corresponding precursor ion in the CSF sample was not intense enough to yield product ions. In this case, the mass accuracy and similarity of the derivatization pattern between the standard and CSF mass spectra were used for identification.

b No standard was available; identification is based on the mass accuracy and similarity of the derivatization pattern.

c Additional details are available in Figure S8. Abbreviations: 3-MT, 3-methoxytyramine; 3-OMD, 3-*O*-methyldopa; 5-HIAA, 5-hydroxyindoleacetic acid; 5-HT, 5-hydroxytryptamine (serotonin); HVA, homovanillic acid; DOPAC, 3,4-dihydroxyphenylacetic acid; DOPAL, 3,4-dihydroxyphenylacetaldehyde; DOPEG, 3,4-dihydroxyphenylglycol; GABA, γ-aminobutyric acid.

Among the identified molecules, a number of amino acids were found in the CSF samples (Figure S4). Amino acid metabolism plays a key role in neurotransmitter synthesis and multiple pathophysiological processes in the brain.^29^

Information about the CSF metabolome is crucial for identifying neurological phenotypes.^30^ Therefore, the rapid detection of multiple neurotransmitters and metabolic intermediates is of high importance. The derivatization approach used in this study allowed for the detection of low-abundance metabolites and those with poor ionization efficiencies. Among the detected molecules, metabolites of the catecholaminergic and serotonergic pathways, trace amines (tyramine), and energy metabolism products, such as creatine and creatinine, were found in the CSF (Figure S5).

The dopamine precursor l-3,4-dihydroxyphenylalanine (l-dopa), which we have previously imaged in tissue sections of l-dopa-treated primate and rodent brains,^23^ was not detected in the CSF samples. However, a metabolic product of l-dopa, 3-*O*-methyldopa (3-OMD), was identified by MS/MS in the CSF samples, and its product ion spectrum was compared with that of a synthetic standard of 3-OMD. 3-OMD was only found in a small number of the analyzed samples (in both technical duplicates) independently of the PD and control groups (Figure S6). Structural identification and differentiation of 3-OMD compared to its isobaric metabolite 3,4-dihydroxyphenylalanine methyl ester (DHPME) were achieved based on MS/MS spectra and the unique derivatization patterns of the two molecules (Figure S6). The two metabolites contain different potential derivatization sites (two for 3-OMD and three for DHMPE) (Figure S6), and the triply derivatized species was not detected in the CSF. MS/MS analysis of CSF and standards for both 3-OMD and DHPME revealed a higher similarity to the spectra of 3-OMD (Figure S6), confirming that the detected metabolite was 3-OMD.

### PD Metabolic Alterations in CSF

**Figure 1:**
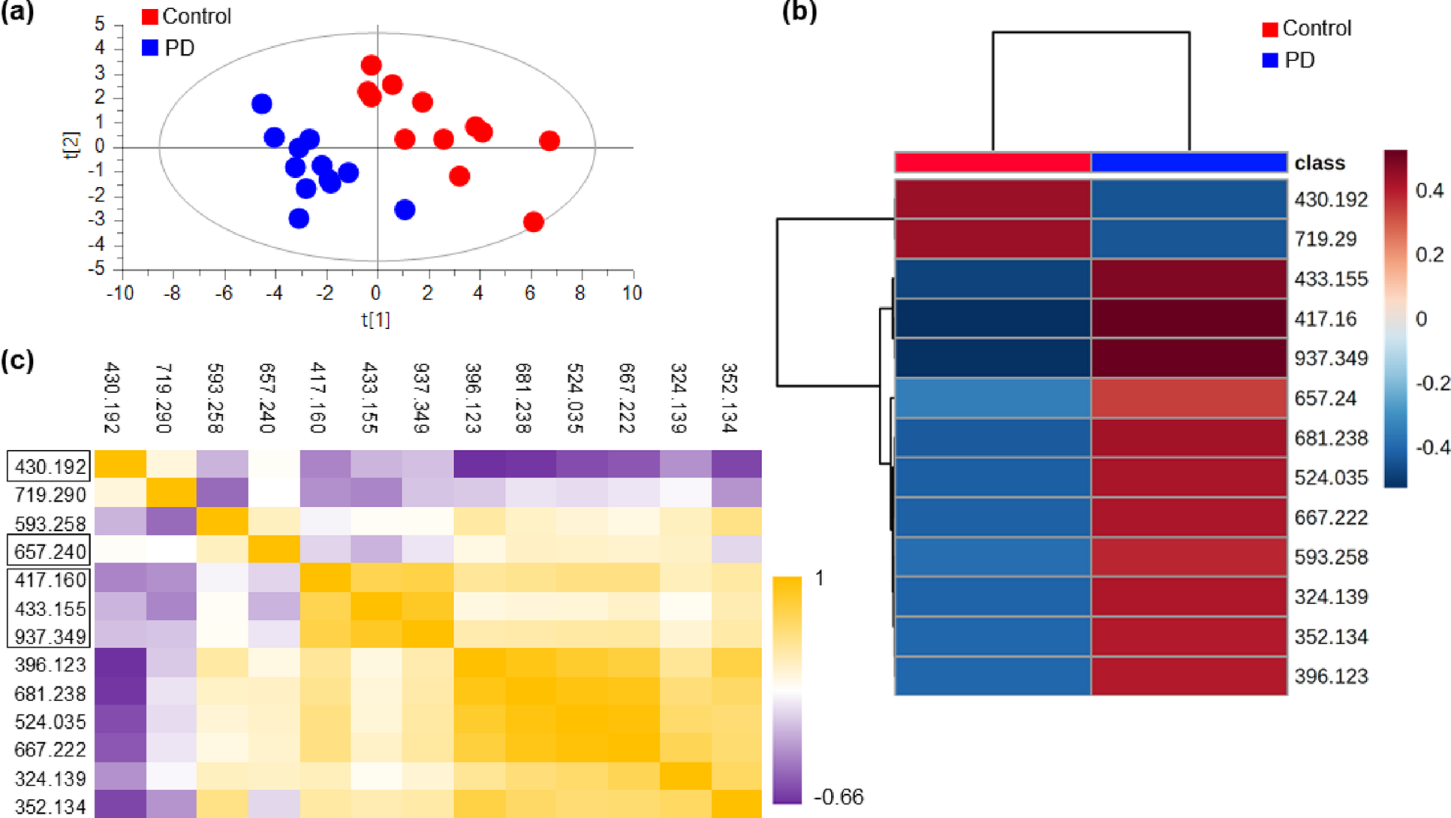
Modeling of PD-induced metabolic alterations in CSF. (a) Score plot of the two principal components of the PLS-DA derived after variable selection. (b) Showing the effect of the significant *m*/*z* values on the two investigated groups. Negative values in the color scale indicate a decrease, whereas positive values indicate an increase. (c) Correlation matrix based on Spearman’s correlation coefficient (*r*) of the significant *m*/*z* values; *P* < 0.05 (two-tailed unpaired *t-*test). Identified metabolites are indicated with rectangles, i.e., norcotinine (*m*/*z* 430.192), hypoxanthine (*m*/*z* 657.240), and aminochromes (*m*/*z* 417.160, 937.349, single- and triple-derivatized, respectively, and *m*/*z* 433.155). Additional information regarding molecule identification confidence and the total number of identifications can be found in Table 1 and Data S1.

Partial least-squares discriminant analysis (PLS-DA), a projection-based method, was performed on the data based on all *m*/*z* values present in the optimized PCA model (i.e., 1937 *m*/*z* values) and considering the first set of technical replicates as the “training set”. The method was considered appropriate owing to the limited sample size of the data set, offering the potential of examining the simultaneous and combined impact of thousands of variables (metabolites).^26^ However, as PLS-DA may be susceptible to bias and overfitting, the derived models were extensively validated and the highlighted metabolites were further investigated.^31,32^ Since the initial model showed very poor predictability (*R*^2^ = 0.883, *Q*^2^ = 0.347), variable selection was performed to identify peaks significantly altered between the two groups, i.e., control and *de novo* PD. The process generated a two-component PLS-DA model with sufficient predictability (*R*^2^ = 0.862, *Q*^2^ = 0.736) including 32 original variables, i.e., *m*/*z* values (Figure 1a). The classification rate was very high (100%), and validation with cross-validated analysis of variance (ANOVA) showed that the model was highly significant (*P* = 3.7 × 10^–5^). The model was also validated through permutation tests and cross-validated scores, all showing good predictability (Figure S7).

The 32 identified *m*/*z* values were inspected in the MSI data to remove false positives/noise, and their significance was evaluated by *t*-test using the average value between the two technical duplicates. Eleven *m*/*z* values (Table S4) were found to be of statistical significance (*P* < 0.05, FDR < 0.1), while hypoxanthine (double-derivatized, *m*/*z* 657.240) demonstrated a strong trend (*P* = 0.093). The abundance of two molecular species was decreased in PD, whereas the abundance of the others increased (Figure 1b). Strong correlations were detected for a few molecules (Spearman’s *r* > 0.9), indicating potential involvement in the same metabolic pathway or peaks deriving from the same species (Figure 1c).

Molecular species with *m*/*z* values of 417.160, 937.349, and 433.1559 displayed strong intercorrelations in the CSF samples (Figure 1c). A standard solution of leukoaminochrome was derivatized and analyzed to assess the presence of the anticipated single (*m*/*z* 419.175), double (*m*/*z* 672.265 and 686.280), and triple (*m*/*z* 925.354, 939.369, and 953.385) derivatized species. In addition to the anticipated species, we also detected dehydrogenated species (*m*/*z* 417.160 and 937.349) and subsequently hydroxylated species (*m*/*z* 433.155) resulting from the derivatization of leukoaminochrome. Further, MALDI-MSI analysis of CSF samples spiked with different concentrations of leukoaminochrome standard demonstrated that in the case of the single derivatized species, the average intensity of *m*/*z* 417.160 was significantly higher than the expected *m*/*z* 419.175. In addition, thorough structural validation of these molecules by MS/MS indicated the presence of a dihydroxyindole ring, a common moiety in dopamine oxidative products,^33^ such as leukoaminochrome and neuromelanin (Figure S8). Therefore, it is highly likely that the above-mentioned molecules were involved in the neuromelanin pathway. A second cluster of highly correlated *m*/*z* values was also detected (Figure 1c), including *m*/*z* 681.238, 524.035, 667.222, 324.139, 352.134, and 396.123, demonstrating a PD-induced increase in the CSF. The MS/MS spectra of CSF, spatial correlation, and spectral evaluation (Smart Formula, Bruker Daltonics) indicated that these ions corresponded to different degrees of derivatization and hydration of molecular species with chemical formula C_5_H_6_O_5_. Since this molecular formula did not correspond to FMP-10 derivatized metabolites listed in hmdb.ca.,^34^ we believe that it was a decarboxylated/decarbonylated product of hydroxylated acid/lactone resembling a glucose structure (open-chain or cyclic).

The metabolite observed at *m*/*z* 430.192 exhibited a significant decrease in samples from individuals with PD. The molecular formula of the underivatized compound was calculated as C_9_H_10_N_2_O. To validate the structure of the metabolite, capillary electrophoresis coupled to a QTOF mass analyzer (CE–MS) was employed, utilizing an underivatized CSF sample (see the Methods section). The molecular formula and fragmentation pattern of the precursor ion ([M + H]^+^, *m*/*z* 163.087) indicated the presence of norcotinine, a nicotine metabolite, as a possible candidate (Figure S9). The *m*/*z* 163.087 ion was observed at a low abundance, resulting in an MS/MS spectrum that produced two product ions consistent with previously published triple quadrupole MS/MS transitions (*m*/*z* 118.08 and 80.05).^35^ After considering the possibility of norcotinine in the CSF samples, post hoc analysis of the CE–MS data identified mass matches for nicotine metabolites cotinine and hydroxycotinine exclusively in the CSF sample containing the *m*/*z* 430.192 ion detected in the MALDI analysis (Figure S10). This finding provides additional support for the identification of norcotinine as the source of the *m*/*z* 430.192 ion in the MALDI data. Notably, the relative intensities of the three putative analytes observed in the CE–MS data align with published abundances of nicotine metabolites in human urine, that is, norcotinine (1–2%), cotinine (75%), and hydroxycotinine (33–40%).^36^ In-source decay fragmentation of the more abundant cotinine and hydroxycotinine further confirmed the presence of product ions consistent with published MS/MS product ions^37^ (Figure S10).

To investigate this hypothesis, the samples were examined based on tobacco use data. Among the control samples, 66.7% were identified as active tobacco users according to their medical records at the time of the CSF sampling, whereas only 8.3% of the *de novo* PD group were tobacco users (Figure S11). Based on this, the impact of tobacco use on the *m*/*z* values, which were identified as significantly different between the control and PD samples, was analyzed (Figure S11). The analysis revealed a clear and highly significant effect on *m*/*z* 430.192 (Figure S11), supporting the hypothesis that the molecule originates from nicotine metabolism. Moreover, an untargeted analysis was performed to support the findings (Table S5). However, due to the higher prevalence of tobacco users in the control group compared to the PD patients, tobacco use emerges as a confounding factor with a strong influence on the given sample set.

## Discussion

In the present study, we developed and validated a method for rapid metabolic profiling of human CSF. By using only 1 μL of CSF without any sample purification and a data acquisition time of less than 4 h, we were able to detect neurochemical alterations between *de novo* PD CSF samples and controls. The applicability of the present method to CSF samples is of particular importance in the field of neurological biomarker discovery. The CSF metabolome reflects the CNS pathology more accurately than the plasma metabolome, although the latter is usually more easily collected, and thus more frequently used in PD biomarker research.^5,9^ We measured the LOD for several isotope-labeled neurotransmitters and demonstrated that the method offered efficient detection and analysis of many metabolites in a small volume of CSF.

The presented method enabled the detection of a wide range of endogenous CSF metabolites with a high mass accuracy. Numerous amino acids were detected as well as neurotransmitters and their metabolic products and metabolic intermediates. Conventionally, catecholaminergic metabolites are detected using electrochemical detectors but with considerably longer analysis time and without simultaneously detecting other metabolic classes.^32^ Previously, we demonstrated comprehensive mapping of these metabolic classes in the brain tissue from human and animal models.^21−24^ In this study, we applied the method for the simultaneous metabolic profiling of biofluids, such as CSF.

Although a number of clinical studies have reported PD-specific metabolic changes using several analytical platforms, the results were inconclusive and inconsistent, highlighting the complexity of the disease and interindividual variability. In addition, the presence of PD-related syndromes, as well as PD-treatment medications, hinders comprehensive metabolic profiling of the disease. However, most studies have found links to oxidative stress and mitochondrial dysfunction.^5,32,38−41^ A crucial response to oxidative stress is altered purine metabolism, and several studies have reported PD-induced changes in purine metabolism.^5,41,42^ Accordingly, we detected altered CSF levels of hypoxanthine in *de novo* PD patients compared with the controls. Nevertheless, a higher statistical power, i.e., a larger population, is required to validate this finding (*P* = 0.092). We also detected that neuromelanin-related species were increased in the CSF of *de novo* PD patients. The oxidation of dopamine to neuromelanin is a physiological process that can generate neurotoxic byproducts causing mitochondrial dysfunction.^38−40^ Indeed, dopaminergic loss in neuromelanin-containing neurons of the substantia nigra is the main neuropathological hallmark of PD. However, the mechanism behind the mitochondrial dysfunction remains unclear, although it has been suggested that it might involve incomplete reduction of aminochrome to leukoaminochrome.^38−40^

The small sample size of the MALDI-MSI study presented here was the main limitation preventing conclusive extrapolation of the findings. Moreover, the implication of tobacco use as a confounding factor raised further questions regarding the interpretability of the results. However, the method showed significant potential for implementation in large cohort studies in a high-throughput fashion. Another limitation was the complexity of the FMP-10 derivatization for the high-confidence identification of metabolites in untargeted studies. Nevertheless, the acquisition of numerous tandem mass spectra may allow the construction of an efficient database for rapid and robust metabolite identification. Therefore, this study served as a valuable approach for establishing a robust multifactorial method for further studies.

## Conclusions

We developed a novel MALDI-MSI method that allowed untreated human CSF samples to be analyzed with a higher throughput protocol, less sample preparation, and shorter data acquisition time compared with previous methods. The method enabled the detection of *de novo* PD-specific metabolic profiles. Our approach could facilitate investigations into how various neurological diseases can be diagnosed and provide improved insights into fundamental neurological processes and disease states.

## Methods

### Chemicals

Deuterated standards, dopamine-*d*_4_ (DA-*d*_4_), norepinephrine-*d*_6_ (NE-*d*_6_), homovanillic acid-*d*_5_ (HVA-*d*_5_), 3,4-*d*_3_dihydroxyphenylalanine (l-dopa-*d*_3_), *d*_4_-3-methoxytyramine (3-MT-*d*_4_), 5-hydroxytryptamine-*d*_4_ (5-HT-*d*_4_), and γ-aminobutyric acid-*d*_6_ (GABA-*d*_6_), were purchased from CDN Isotopes (Quebec, Canada). All standards used for identifying analytes by MS/MS and 97% triethylamine, trifluoroacetic acid, and sodium hydroxide flakes were purchased from Merck KGaA (Darmstadt, Germany). Water, methanol, isopropanol, and acetonitrile were of HPLC grade (VWR, Stockholm, Sweden). Formic acid was LC–MS grade (Thermo Fisher Scientific, Göteborg, Sweden). The reactive matrix FMP-10 was purchased from Tag-ON AB (Uppsala, Sweden).^21,22^

### Limit of Detection of Isotope-Labeled Neurotransmitters in CSF

LOD values for different neurotransmitters in the CSF were estimated by spiking a control CSF sample with isotope-labeled neurotransmitters at different concentrations. Stock solutions of HVA-*d*_5_, l-dopa-*d*_3_, 5-HT-*d*_4_, DA-*d*_4_, NE-*d*_6_, 3-MT-*d*_4_, and GABA-*d*_6_ were prepared at 1 mg/mL in 100% ethanol, except GABA-*d*_6_, which was dissolved in water (HPLC grade). Serial dilutions of the isotope-labeled neurotransmitters were prepared in CSF at the following concentrations: 10, 3.3, 1.1, 0.37, 0.12, 0.04, and 0 ng/μL. Aliquots of 1 μL of spiked and control CSF were spotted onto a stainless steel MALDI target plate and left to dry at room temperature for about 30 min. For GABA-*d*_6_ and DA-*d*_4_, all concentrations were spotted in triplicate. The derivatizing MALDI matrix FMP-10 was prepared as previously described.^21,22^ In brief, 20 passes of 4.4 mM FMP-10 in 70% acetonitrile were sprayed pneumatically (nitrogen pressure of 6 psi) in horizontal lines over the CSF spots using a TM Sprayer (HTX Technologies) with a solvent flow rate of 80 μL/min, nozzle temperature of 90 °C, nozzle velocity of 1100 mm/min, and track spacing of 2 mm.

Data were acquired as a MALDI-MSI data set (using the MS acquisition parameters detailed below) by collecting data from the spots at a lateral resolution of 200 μm. Raw data were imported into msIQuant,^43^ and the average intensity values from all spiked CSF spots and controls were extracted to obtain calibration curves for all isotope-labeled standards. The LOD for all compounds was calculated using the LINEST function based on the expression LOD = 3.3*x* σ/*S*, where *S* is the slope of the calibration curve and σ is the standard deviation of the *y* intercept.

### Clinical CSF Samples

CSF samples were obtained from Karolinska Institutet (Stockholm, Sweden). All investigations were performed in agreement with the Helsinki Declaration and with permission from the local ethical committee of the hospital (no. 2019-04967). A total of 24 CSF samples were obtained from patients with *de novo* PD (*n* = 12) and control subjects (*n* = 12). Control CSF samples were collected from patients who were being investigated for other complaints; none of these patients had any clinical or laboratory signs of severe condition. CSF was acquired by lumbar puncture and collected in polypropylene tubes, which were subsequently centrifuged (1300–1800*g*, 4 °C, 10 min). The supernatant was carefully removed and dispensed in 500 μL volumes into glass tubes before storage at −80 °C. The time between collection and freezing was less than 60 min. Samples were subsequently thawed on ice, and 1 μL of each CSF sample was spotted in duplicate onto a stainless steel MALDI target plate in a randomized order. A QC sample was prepared as a pool of equal volumes (3 μL) of all CSF samples and was spotted at a volume of 1 μL per spot in nine different spots on the same MALDI target plate. The QC was used to monitor the experimental procedure and quality of the results. All samples were left to dry for 30 min. FMP-10 was sprayed over the spots, as described above. After FMP-10 application, the spotted MALDI target plate was desiccated at room temperature for 15 min prior to scanning on a flatbed scanner (Epson Perfection V500, Japan).

### MALDI-MSI Analysis

All MALDI-MSI experiments were performed by using a MALDI-FTICR-MS instrument (Solarix XR 7T-2Ω, Bruker Daltonics, Bremen) equipped with a Smartbeam II 2 kHz Nd:YAG laser. Acquisitions were set up by using ftmsControl (Bruker Daltonics, Bremen, Germany) and flexImaging (Bruker Daltonics, Bremen, Germany). The laser power was optimized at the start of each analysis. Samples were analyzed in the positive ion mode using the quadrature phase detection (QPD) (2ω) mode over a mass range of *m*/*z* 150–1000 providing a mass resolution of about 220,000 at *m*/*z* 400. The medium laser focus setting was used for acquisitions at a spatial resolution of 200 μm, and the laser power was optimized prior to acquisition. Spectra were recorded by summing signals from 100 laser shots per pixel. The quadrupole isolation *m*/*z* (Q1) was set at *m*/*z* 379. The time-of-flight (TOF) and transfer optics frequency values were adjusted to 0.700 ms and 4 MHz, respectively. A matrix-derived peak at *m*/*z* 555.223 was used as a lock mass for internal *m*/z calibration. Red phosphorus was used for the external calibration of the method. Samples were analyzed in a random order to prevent bias due to possible matrix vacuum instability or changes in the mass spectrometer’s sensitivity.

### Peak Intensity Acquisition

A list of peaks was generated in the SCiLS Lab GUI (Bruker Daltonics) from centroid peak picked data (SQLite format) by intensity thresholding. This extracted table contained approximately 2900 peaks as rows and 57 analyzed samples as columns, including nine QC and two technical replicates from each of the 24 CSF samples (Data S3). For each sample and peak, pixels making up the spot were collected through SCiLS API and stripped of missing values, and the median of the remaining values was recorded as the intensity for each specific sample and *m*/*z* value.

### Data Analysis

If no sample purification is performed, then MSI data can include a significant amount of noise. Therefore, filtering of the extracted features was performed based on the correlation of their *m*/*z* values (*m*/*z* ≥ 300) to known FMP-10 matrix-derived peaks (peaks with correlation coefficient |*r*| > 0.8 with *m*/*z* 555.223 were removed), their dependence on the experimental run order, and their variance (>50% CV) in the QC samples. Multivariate statistics were calculated using SIMCA software (Sartorius Stedim Biotech, Umeå, Sweden, version 15.0). Prior to analysis, variables were autoscaled (mean-centered and divided by the respective standard deviation). PCA was performed by considering all columns of the table as *X* variables to obtain an initial overview of the data. PCA is a projection method resulting in dimensional reduction. The principal components (PCs) derived through projection represent new (latent) variables that summarize the information included in the initial set of variables, in this case, *m*/*z* values. A strong outlier belonging to the second set of duplicates located outside the Hotelling T2 ellipse was detected, which was identified as an experimental error and therefore removed (Figure S1). PCA after the outlier removal revealed high interindividual variability of the human CSF samples, as expected (Figure S1). Because the score values of the first and second PC indicated some differences between the first and second sets of technical replicates (compared as pairs), *m*/*z* values significantly different between the two replicates were removed. This process reduced the score difference between the first and the second replicates by 2.6% for the first PC and 60% for the second PC. The 10-component derived PCA was used for further model development. The QC samples showed denser clustering than the individual CSF samples and were located close to the center of the axes formed by the first (23% variance explained) and second (13% variance explained) PCs.

PLS-DA, a supervised classification extension of PCA, was applied to construct the prediction models. The variable selection was based on values of the variable influence to projection (VIP), the weight (*w*) in the loading plot, and the size of coefficients. Variables with VIP < 1, low weight in the loading plot, or low coefficient were excluded. The predictive ability and robustness of the model were first evaluated using cross-validation as internal validation according to the 7-fold option of Simca-P. Robust models should demonstrate a difference between *R*^2^ and the cross-validated *R*^2^, i.e., *Q*^2^, lower than 0.2. Cross-validation ANOVA was also applied, with *P* < 0.05 indicating a robust model. Permutation tests (100 permutations) were performed by randomly reordering the response variables, and the newly derived *R*^2^ and *Q*^2^ values were plotted against the degree of correlation between the permuted and original data. Finally, the models were validated based on their cross-validated scores (CV scores) and through external validation, specifically employing the second set of technical duplicates as a test set. The results demonstrated an acceptable level of predictability, with a classification rate of 65% (out of the 23 predicted classes, 15 were correctly predicted, 5 were classified as borderline, and 3 were mispredicted).

### Metabolite Identification

The extracted peak list was matched to the Human Metabolome Database (www.hmdb.ca)^34^ using a custom script written in the Python programming language version 3.7 (www.python.org). No deisotoping was performed. Initially, a recalibration of the data set was performed based on five known peaks spread across the spectra using the Python Scipy function fmin (quadratic fit). The script then included an identification algorithm that disqualified any matches deviating by more than 1.25 ppm from the theoretical mass with FMP-10. The *m*/*z* ratio matching was streamlined with Python’s bisect module using the bisect_left function. Matching metabolites that did not include either a primary amine or phenolic group were disqualified as FMP-10 has only been shown to derivatize molecules with these functional groups.^21^ Last, molecules with a single functional group derivatized by FMP-10 were disqualified from being considered double- or triple-derivatized. When it was possible to isolate the parent ion directly from the derivatized CSF samples, MS/MS spectra were obtained and compared with the spectra of FMP-10 derivatized standard compounds. For MS/MS, a 1 Da isolation window was used, and different collision energy voltages were tested (from 20 to 45 V). The use of different collision energies assisted discrimination of the targeted *m*/*z* peak from neighboring peaks. To identify metabolites with high importance for the separation of the two groups, tandem mass spectrometry was used to investigate the fragmentation patterns of the unknown metabolites.

### CE–MS Analysis

Capillary electrophoresis separations were conducted by using 100 cm bare fused silica capillaries. The background electrolyte consisted of 1% formic acid, and gas phase ions were generated using a previously described coaxial sheath flow CE-ESI interface.^44^ Hydrodynamic injection of unprocessed CSF was employed to introduce a sample volume of 6–19 nL into the separation capillary. The separation potential was set at 26–27 kV, ensuring a stable current of ∼7 μA. The generated ions were analyzed using a Synapt G2Si instrument (Waters Corp., Manchester, UK) operating in positive ion mode, resolution mode, and with or without mobility enabled for MS1 scans. To fragment the *m*/*z* 163.087 ion, high-definition data-dependent acquisition was employed in sensitivity mode with a precursor isolation width of ±0.015 Da and a transfer collision energy ramp of 10–60 eV. The instrument was calibrated with sodium formate ion clusters on the day of use. Internal calibration of acquired mass spectra was performed using leucine–enkephalin in the sheath liquid.

## Supporting Information Available

Overview of data; structural validation of amino acids in CSF samples; structural validation of neurotransmitters and other metabolites; MALDI-MSI of amino acids in CSF samples from PD patients and controls; MALDI-MSI of neurotransmitters and metabolites in CSF samples from PD patients and healthy controls; isomeric compound identification in CSF patient samples through MALDI-MS/MS analysis; validation of the PLS-DA model; structural validation of neuromelanin-related metabolites; structural validation of *m*/*z* 163.087 with CE–MS/MS; extracted ion electropherograms from CE–MS separations; effect of tobacco use on the investigated CSF metabolites; limit of detection (LOD) of selected deuterated neurotransmitters and metabolites; mass accuracy of isotope-labeled standards and corresponding endogenous NTs obtained from the LOD experiment; list of synthetic standards used for structure validation of CSF detected metabolites and the theoretical *m*/*z* values of all potential derivatization species; univariate statistical validation (two-tailed *t*-test) of the inspected high VIP *m*/*z* values; untargeted univariate statistical analysis (two-tailed *t*-test) of the tobacco use effect (PDF)
Detection of metabolites available in the human metabolome database based on the mass accuracy (XLSX)
MS/MS spectra from selected standards compared to the corresponding MS/MS spectra of CSF (XLSX)
List of peaks generated from centroid peak picked data by intensity thresholding (XLSX)
