# Supplementary material for: Rapid Metabolic Profiling of 1 μL Crude Cerebrospinal Fluid by Matrix-Assisted Laser Desorption/Ionization Mass Spectrometry Imaging Can Differentiate De Novo Parkinson’s Disease
Source: Anal Chem. 2023 Dec 7;95(50):18352–60. doi: 10.1021/acs.analchem.3c02900 (PMC10733901; doi:10.1021/acs.analchem.3c02900)
Supplement: Supplementary file 1 — ac3c02900_si_001.pdf [file ac3c02900_si_001.pdf]

SUPPORTING INFORMATION

Rapid metabolic profiling of one microliter crude CSF by MALDI MS can differentiate de novo Parkinson's disease

Theodosia Vallianatou[1#], Anna Nilsson[1#], Patrik Bjärterot[1], Reza Shariatgorji[1], Nuria Slijkhuis[1†], Jordan T. Aerts[1], Erik T. Jansson[1], Per Svenningsson[2], and Per E. Andrén[1*]

1. Dept. of Pharmaceutical Biosciences, Spatial Mass Spectrometry, Science for Life Laboratory, Uppsala University, SE-75124 Uppsala, Sweden.
2. Dept. of Clinical Neuroscience, Karolinska Institute, SE-17177 Stockholm, Sweden.

#Equal contribution

[*]Corresponding author. Email: per.andren@uu.se

**SUPPORTING FIGURES**		**Page S2-S14**
Figure S1	Overview of data	Page S2
Figure S2	Structural validation of amino acids in CSF samples	Page S3
Figure S3	Structural validation of neurotransmitters and other metabolites	Page S4
Figure S4	MALDI-MSI of amino acids in CSF samples from PD patients and controls	Page S5
Figure S5	MALDI-MSI of neurotransmitters and metabolites in CSF samples from PD patients and healthy controls	Page S6
Figure S6	Isomeric compound identification in CSF patient samples through MALDI-MS/MS analysis	Page S7
Figure S7	Validation of the PLS-DA model	Page S8
Figure S8	Structural validation of neuromelanin-related metabolites	Page S9-S10
Figure S9	Structural validation of *m/z* 163.087 with CE–MS/MS	Page S11
Figure S10	Extracted ion electropherograms from CE–MS separations	Page S12-S13
Figure S11	Effect of tobacco use on the investigated CSF metabolites	Page S14

**SUPPORTING TABLES**		**Page S15-S20**
Table S1	Limit of detection (LOD) of selected deuterated neurotransmitters and metabolites	Page S15
Table S2	Mass accuracy of isotope-labeled standards and corresponding endogenous NTs obtained from the LOD experiment	Page S16
Table S3	List of synthetic standards used for structure validation of CSF detected metabolites and the theoretical *m/z* values of all potential derivatization species.	Page S17-S18
Table S4	Univariate statistical validation (two-tailed *t*-test) of the inspected high VIP *m/z* values	S19
Table S5	Untargeted univariate statistical analysis (two-tailed *t*-test) of the tobacco use effect	S20

Supporting Figures

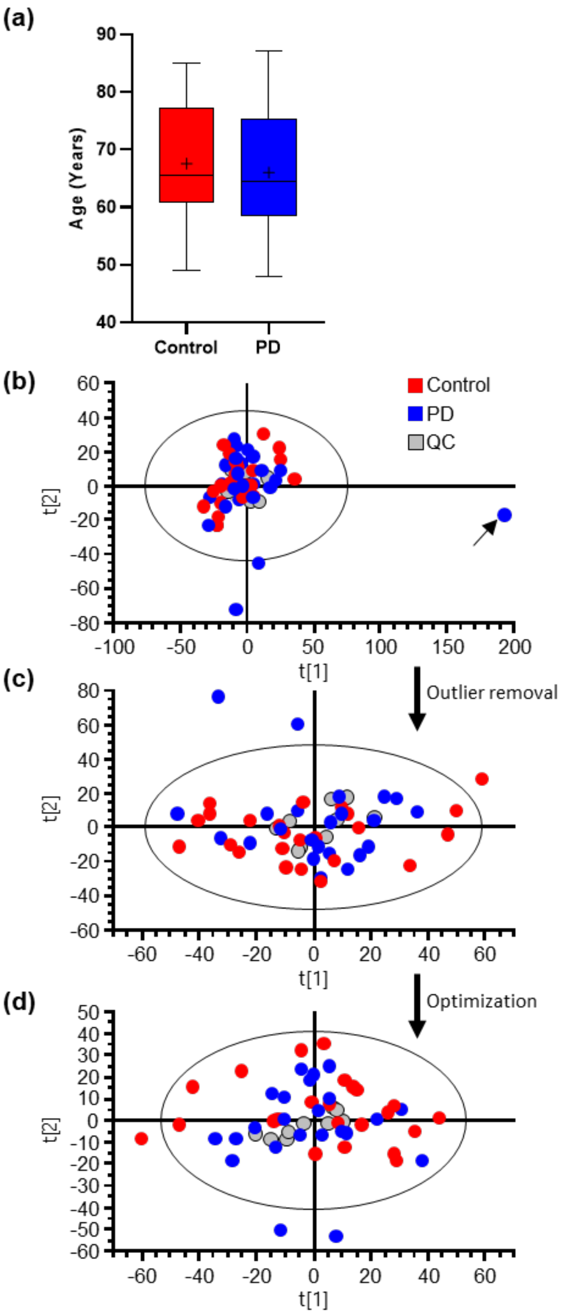

Figure S1. Overview of data.

(a) Box plots of age in the control and PD group. The mean age is indicated by + in each box plot. **(b)** Score plot of the two principal components of PCA including all variables after filtering and both technical replicas. The arrow indicates a strong outlier which was identified as an experimental error. **(c)** Score plot of the two principal components of PCA after the removal of the strong outlier. **(d)** Score plot of the two principal components of PCA after model optimization, i.e., removal of *m*/*z* values significantly different between the first and second technical duplicates. Abbreviations: PD, *de novo* Parkinson's disease patients; QC, quality control.

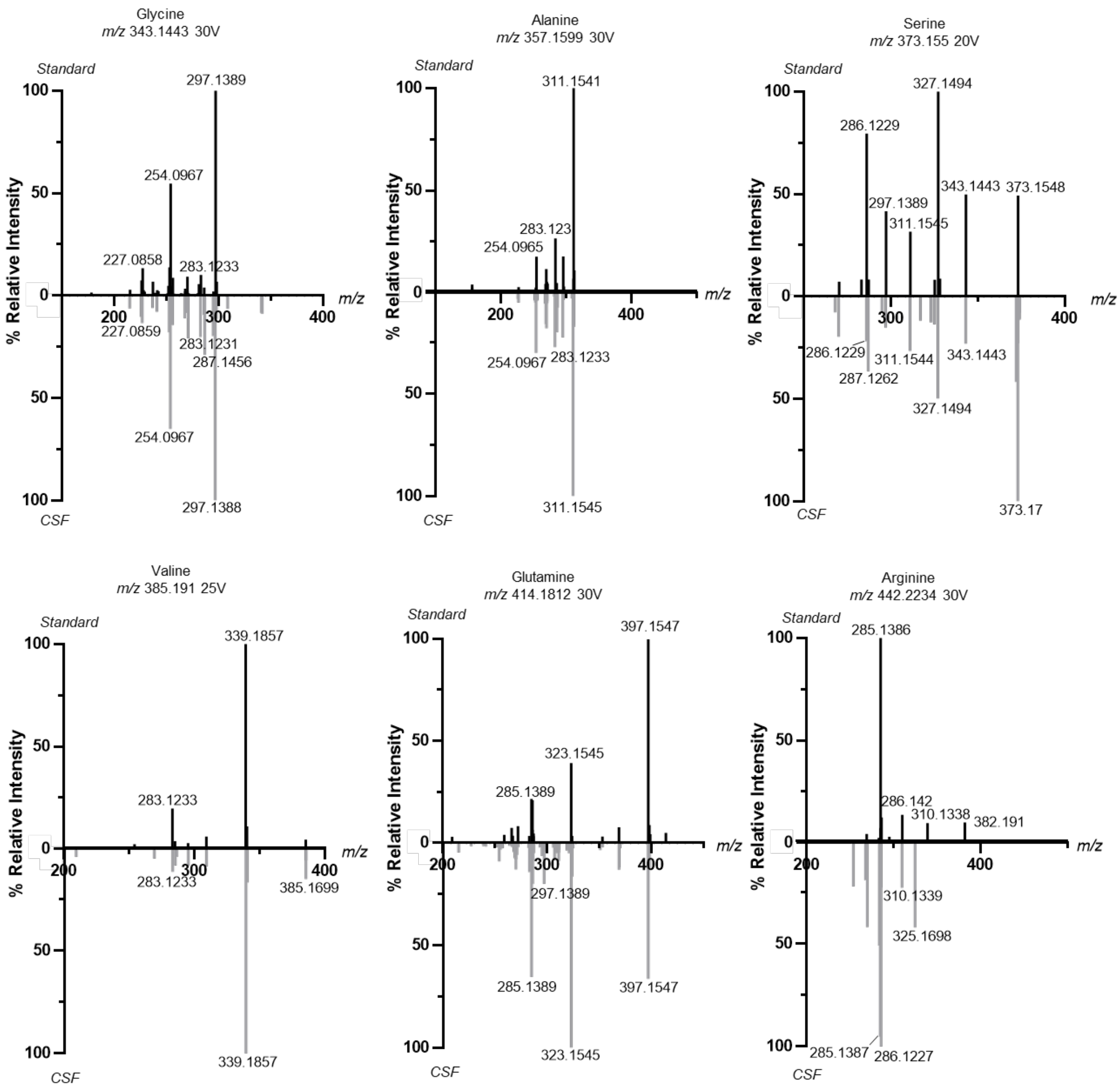

Figure S2. Structural validation of amino acids in CSF samples.

Representative mirror-matched MALDI tandem mass spectra of FMP-10 derivatized analytes in standard (black) and CSF (gray) samples collected with 20-30 V collision energy. Extracted peak lists from the corresponding MS/MS spectra can be found in Supplementary Data 2.

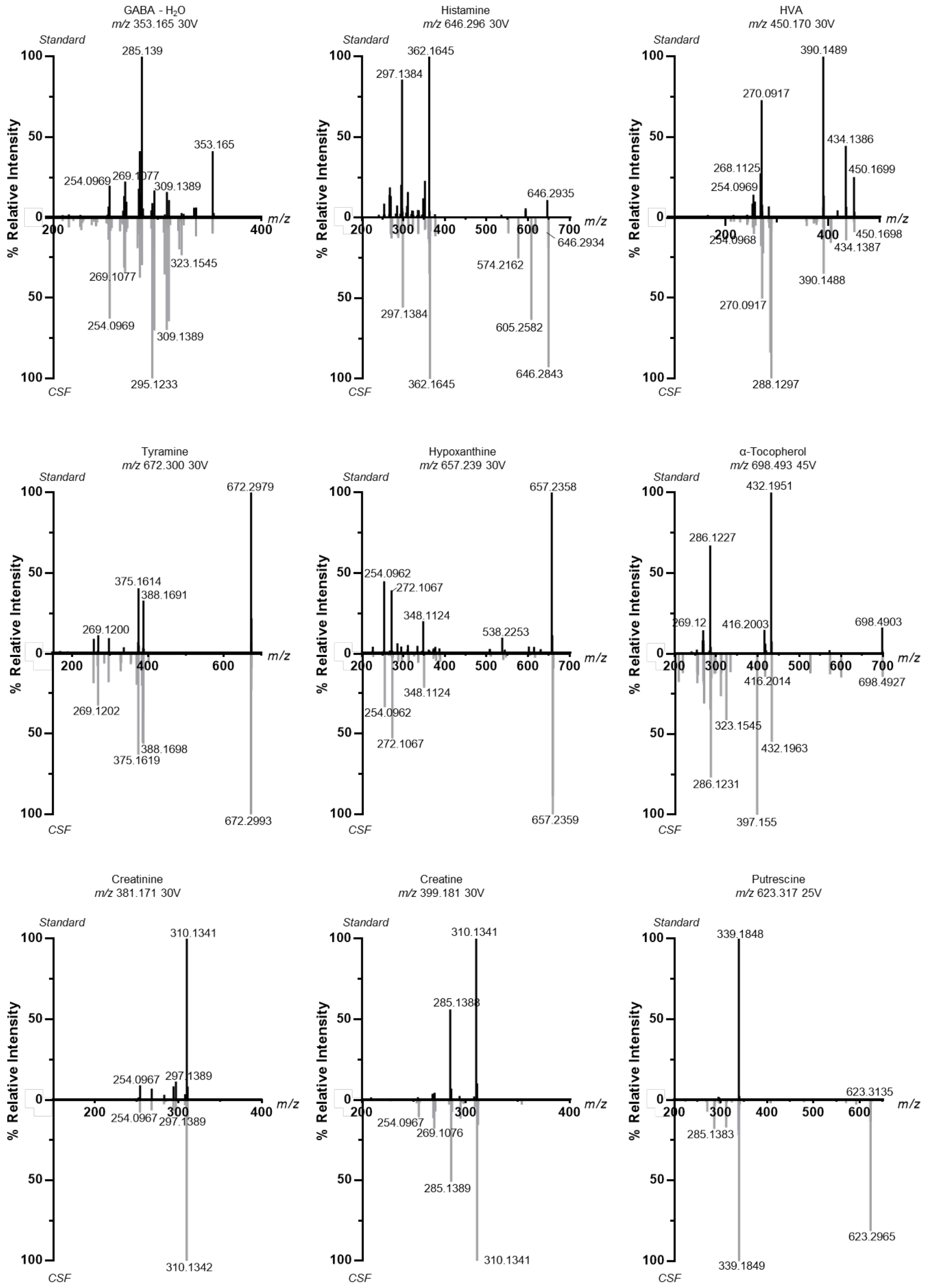

Figure S3. **Structural validation of neurotransmitters and other metabolites.**

Representative mirror-matched MALDI tandem mass spectra of FMP-10 derivatized analytes in standard (black) and CSF (gray) samples collected with 20-30 V collision energy. Extracted peak lists from the corresponding MS/MS spectra can be found in Supplementary Data 2.

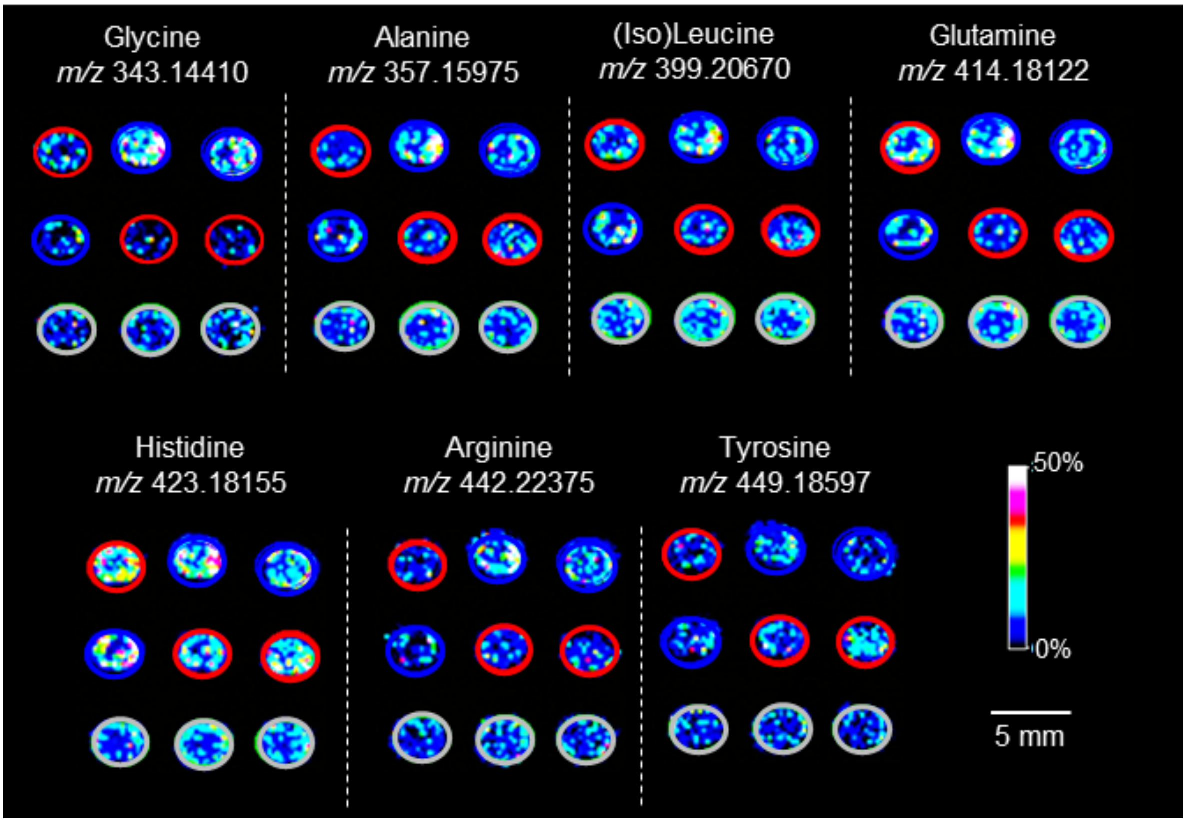

Figure S4. MALDI-MSI of amino acids in CSF samples from PD patients and controls.

MALDI-MSI of selected FMP-10 derivatized neurotransmitters and metabolites in representative CSF spots from PD (blue), control (red) and quality control (grey) samples. All MSI data are normalized by root mean square (RMS) and scaled to 50% of the maximum intensity. Step size 200 x 200 µm, laser size ~80 µm, 180 pixels/spot, runtime ~3 min/spot, mass resolution 220,000 at *m/z* 450. Information concerning molecular identification confidence and total number of identifications can be found in Table 1 and Supplementary Table 3.

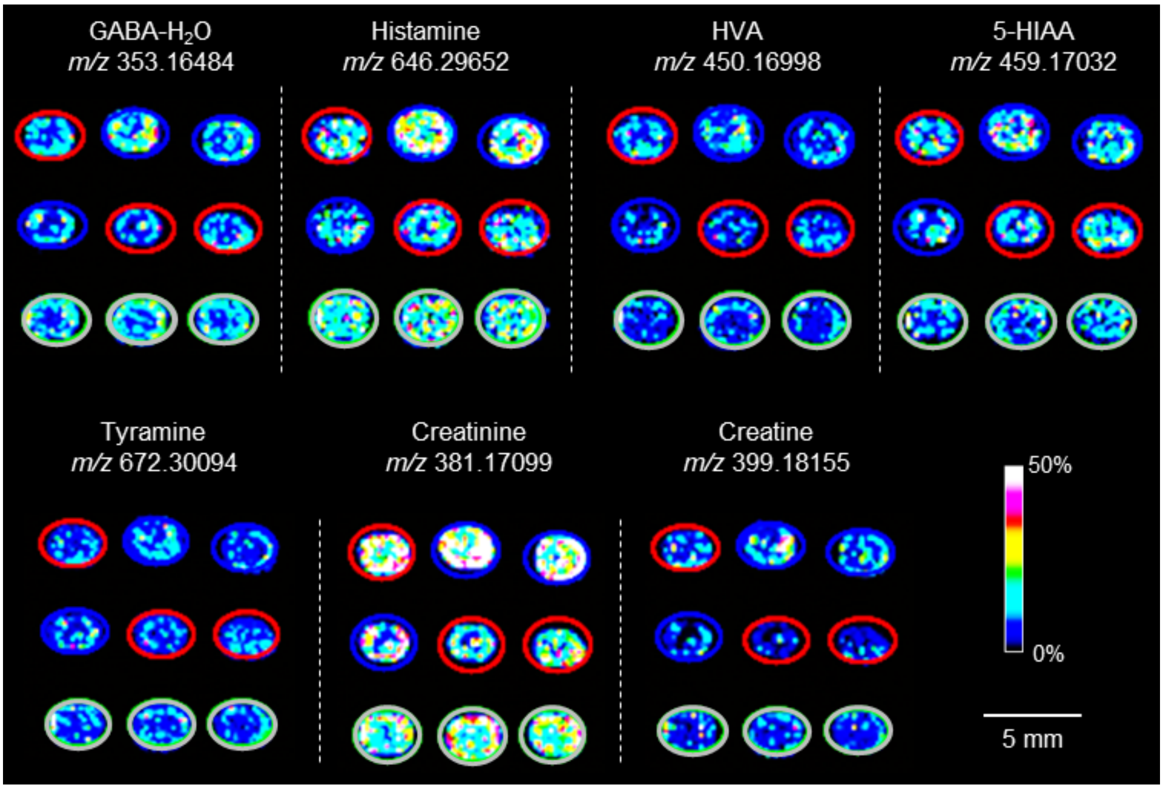

Figure S5. MALDI-MSI of neurotransmitters and metabolites in CSF samples from PD patients and healthy controls.

MALDI-MSI of selected FMP-10 derivatized neurotransmitters and metabolites on representative CSF spots from PD (blue), control (red) and quality control (grey) samples. All MSI data are normalized by root mean square (RMS) and scaled to 50% of the maximum intensity. Step size 200 x 200 µm, laser size ~80 µm, 180 pixels/spot, run time ~3 min/spot, mass resolution 220,000 at *m/z* 450. Information concerning molecular identification confidence and total number of identifications can be found in Table 1 and Supplementary Table 3.

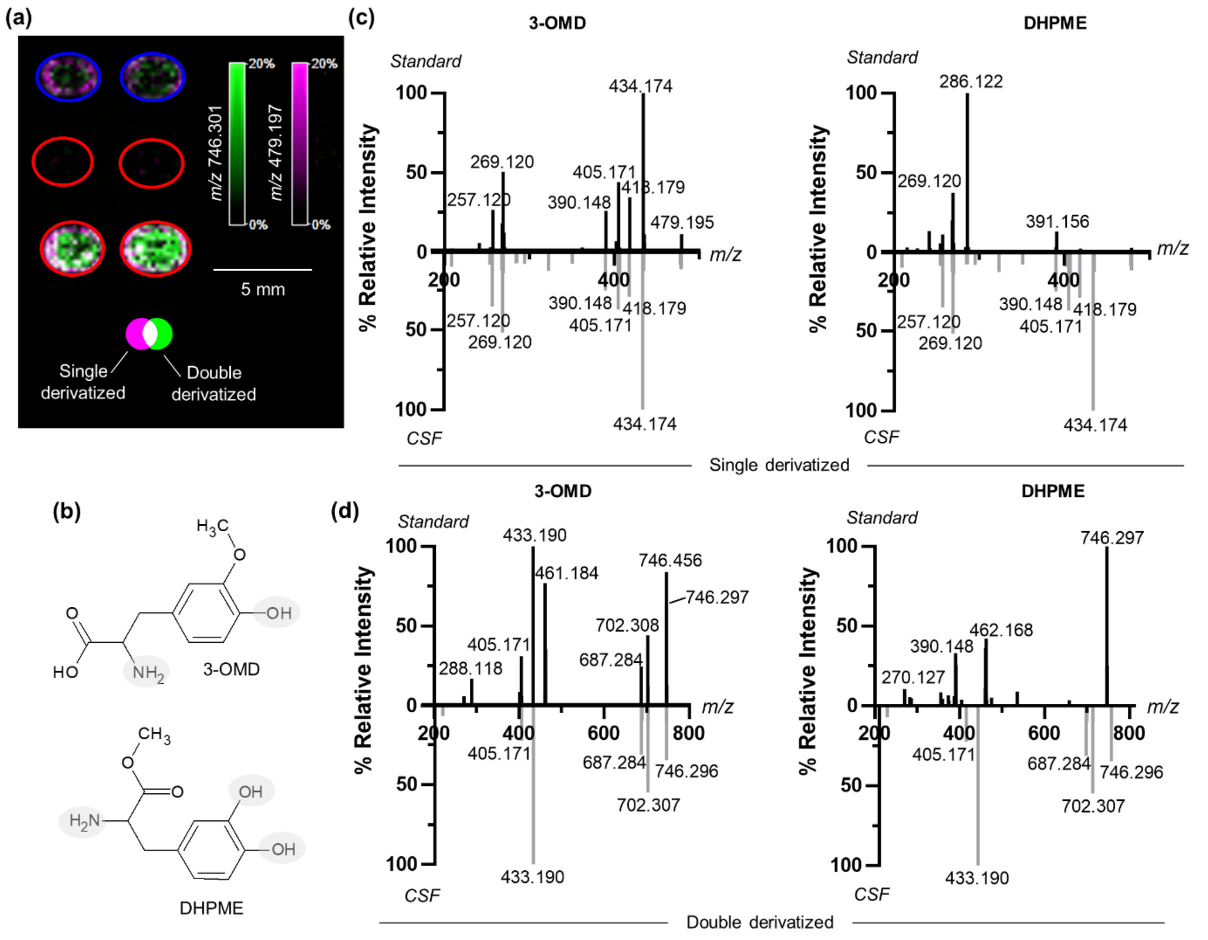

Figure S6. Isomeric compound identification in CSF patient samples through MALDI-MS/MS analysis.

Employing MALDI-MS/MS, isomeric compounds are delineated within CSF samples obtained from patients. This approach facilitated the precise characterization and differentiation of the L-dopa metabolite, 3-O-methyldopa (3-OMD), and its isobaric analog, the precursor of L-dopa, 3,4-dihydroxyphenylalanine methyl ester (DHPME). The differentiation was based on thorough examination of MS/MS spectra in conjunction and the unique derivatization patterns of the two molecules. **(a)** Overlaid MALDI-MSI of *m/z* 479.197 (magenta, scaled to 20 % of maximum intensity) and *m/z* 746.301 (green, scaled to 20 % of maximum intensity) in CSF samples from control (circled in red) and PD (circled in blue) patients. (**b**) Structures of 3-OMD and DHPME. Derivatization sites are highlighted by gray circles. (**c**) Mirror-matched MALDI MS/MS spectra of FMP-10 single derivatized analytes (*m/z* 479.197) in standard (black) and CSF (gray) samples collected with 1 *m/z* isolation window and 30 V collision energy. (**d**) Mirror-matched MALDI tandem mass spectra of FMP-10 double derivatized analytes (*m/z* 746.301) in standard (black) and CSF (grey) samples collected with 1 *m/z* isolation window and 30 V collision energy. Step size 200 x 200 µm, laser size ~80 µm, 180 pixels/spot, runtime ~3 min/spot, mass resolution 220,000 at *m/z* 450. Extracted peak lists from the corresponding MS/MS spectra can be found in Supplementary Data 2. Abbreviations: 3-OMD, 3-O-methyldopa; DHPME, 3,4- dihydroxyphenylalanine methyl ester.

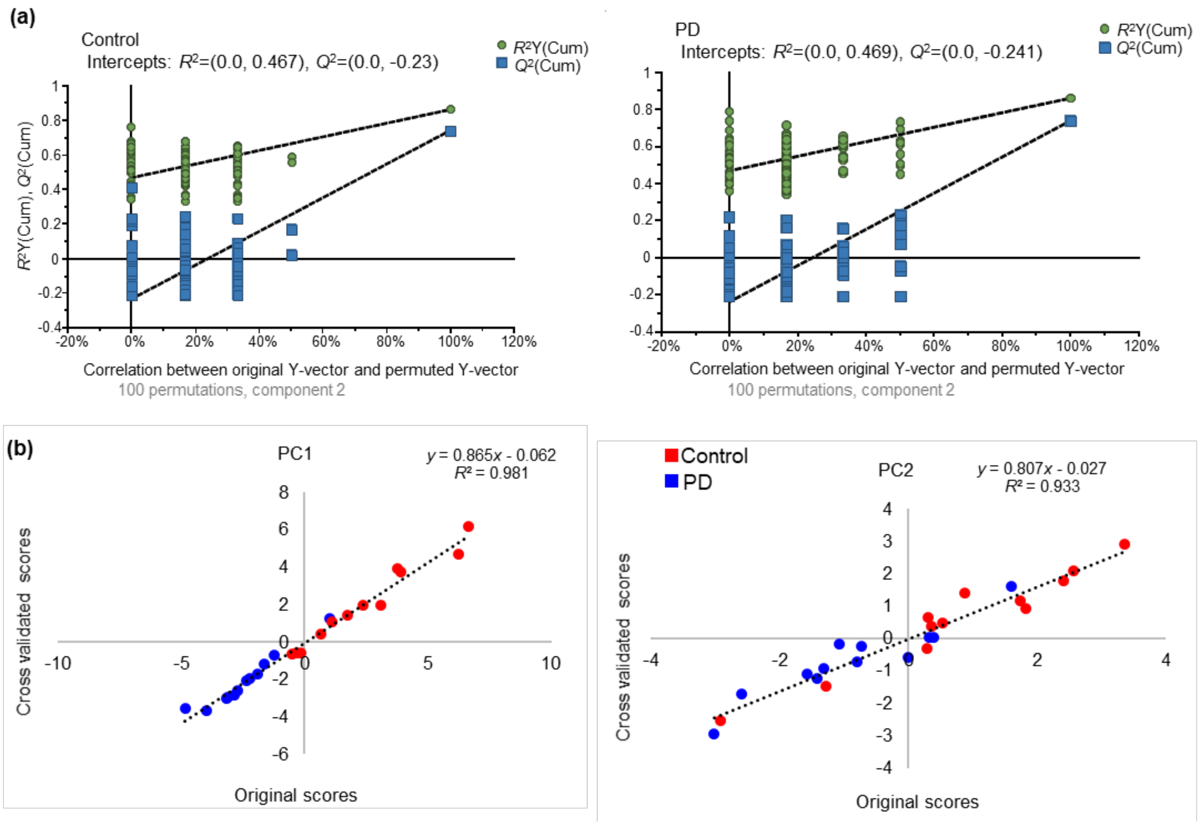

Figure S7. Validation of the PLS-DA model.

(a) Permutation tests calculated from 100 permutations showing the correlation between the original and permuted statistics (R^2, Q^2). The process included random re-ordering of the response variables (i.e., investigated classes), and the newly derived R^2 and Q^2 are plotted against the degree of correlation between the permuted and original data. The intercept is a measure of the overfit. Significantly lower R^2 and Q^2 values compared to the original values indicate the robustness of the model. Negative cumulative Q^2(cum) values indicate that the ratio *PRESS*/*SS*, where *PRESS* is the squared differences between observed and predicted values for the *Y*-data kept out of the model fitting and *SS* is the residual sum of squares of the previous component, is higher than 1. **(b)** Correlation between the original scores of the PLS-DA model and the cross-validated scores for the first (PC1) and second (PC2) components. For both principal components, the correlation between the original and cross-validated scores was linear (R^2>0.9), although better for PC1.

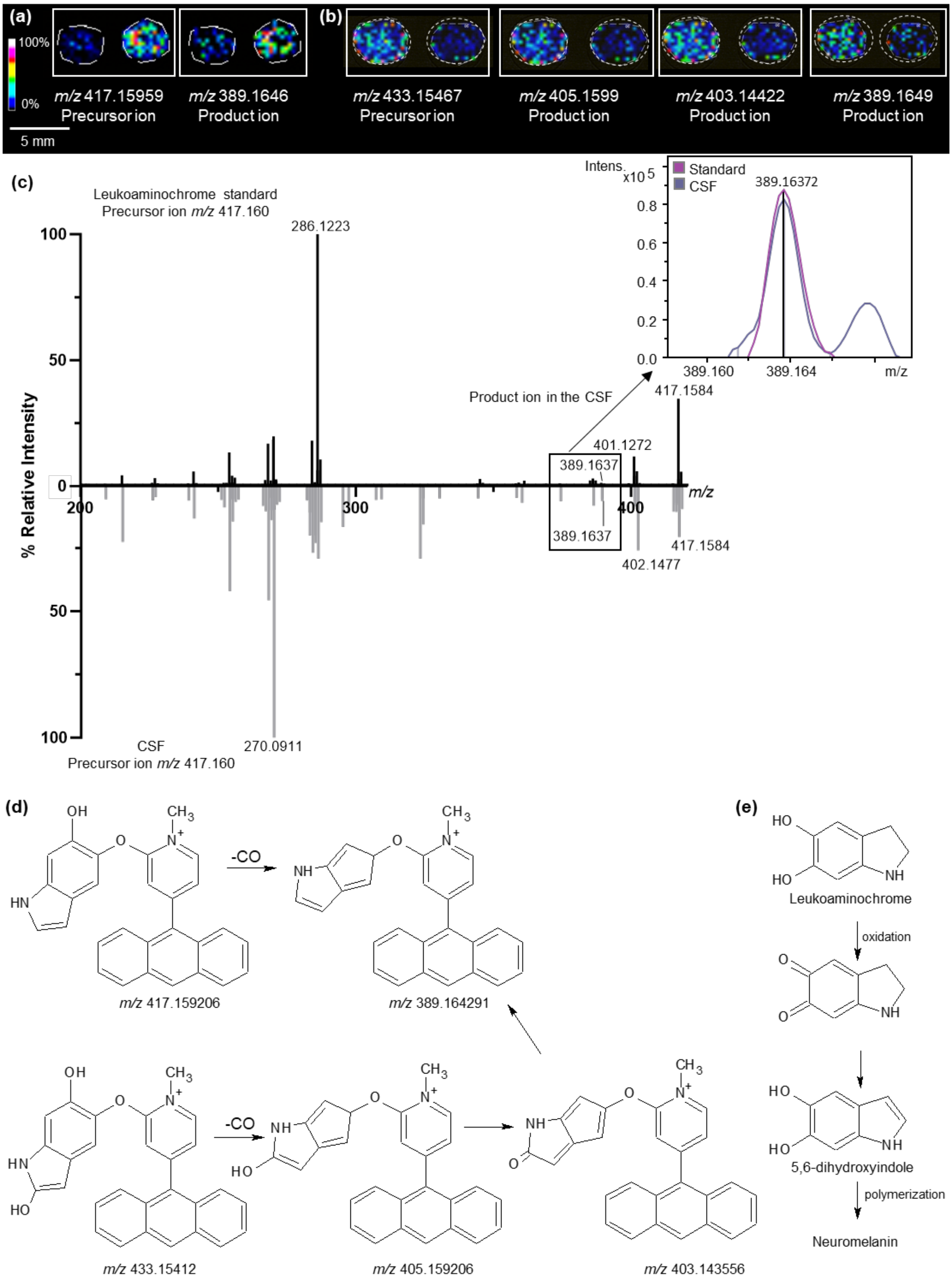

(a)
100%
0%
m/z 417.15959
Precursor ion
m/z 389.1646
Product ion
5 mm
(b)
m/z 433.15467
Precursor ion
m/z 405.1599
Product ion
m/z 403.14422
Product ion
m/z 389.1649
Product ion
(c)
Leukoaminochrome standard
Precursor ion m/z 417.160
286.1223
% Relative Intensity
100
50
0
200
300
400
m/z
Product ion in the CSF
389.1637
401.1272
417.1584
402.1477
270.0911
CSF
Precursor ion m/z 417.160
Intens.
x10 5
Standard
CSF
389.16372
0.8
0.6
0.4
0.2
0.0
389.160
389.164
m/z
(d)
OH
CH3
O
N+
-CO
NH
HN
m/z 417.159206
m/z 389.164291
HO
m/z 433.15412
m/z 405.159206
m/z 403.143556
(e)
HO
NH
Leukoaminochrome
oxidation
O
5,6-dihydroxyindole
polymerization
Neuromelanin

Figure S8. Structural validation of neuromelanin-related metabolites.

(a) MS/MS imaging of precursor ion m/z 417.160, corresponding to the dehydrogenated single derivatized species of leukoaminochrome, also known as 5,6-dihydroxyindole (e), in CSF spots from one control (left) and one PD (right) sample. The product ion (m/z 389.165) exhibited a similar increase in the PD sample as the precursor ion. Step size 250 x 250 µm, laser size ~80 µm, 100 pixels/spot, runtime ~1.5 min/spot, mass resolution ~200,000 at *m/z* 417. **(b)** MS/MS imaging of precursor ion *m/z* 433.155, representing the single derivatized hydroxylated 5,6-dihydroxyindole in CSF spots from one control (left) and one PD (right) sample. The specific product ions (*m/z* 405.160, 403.144, 389.165) showed a similar increase in the PD sample as the precursor ion. All *m/z* images are scaled to 100% max intensity. Step size 200 x 200 µm, laser size ~80 µm, 180 pixels/spot, runtime ~3 min/spot, mass resolution ~200,000 at *m/z* 417. (**c**) Mirror-matched MS/MS spectra of precursor ion *m/z* 417.160 in FMP-10 derivatized leukoaminochrome standard (upper spectrum) and CSF (lower spectrum), collected with 1 *m/z* isolation window and 30 V collision energy. The CSF-specific product ion (m/z 389.164) is highlighted in both MS/MS spectra and is further exemplified in the enlarged, overlaid MS/MS spectra from the standard and CSF samples. The mirror-matched spectra display the % relative intensities of the detected ions within the mass range of m/z 200-420, applying a 1.5 signal-to-noise ratio filter. The enlarged overlaid MS/MS spectra represent arbitrary intensity units. **(d)** Suggested fragmentation pattern of the singly derivatized species *m/z* 417.169 and *m/z* 433.155. The theoretical *m/z* values following derivatization are provided. **(e)** Oxidation of leukoaminochrome results in the CSF-detected species, which subsequently polymerize to form neuromelanin.

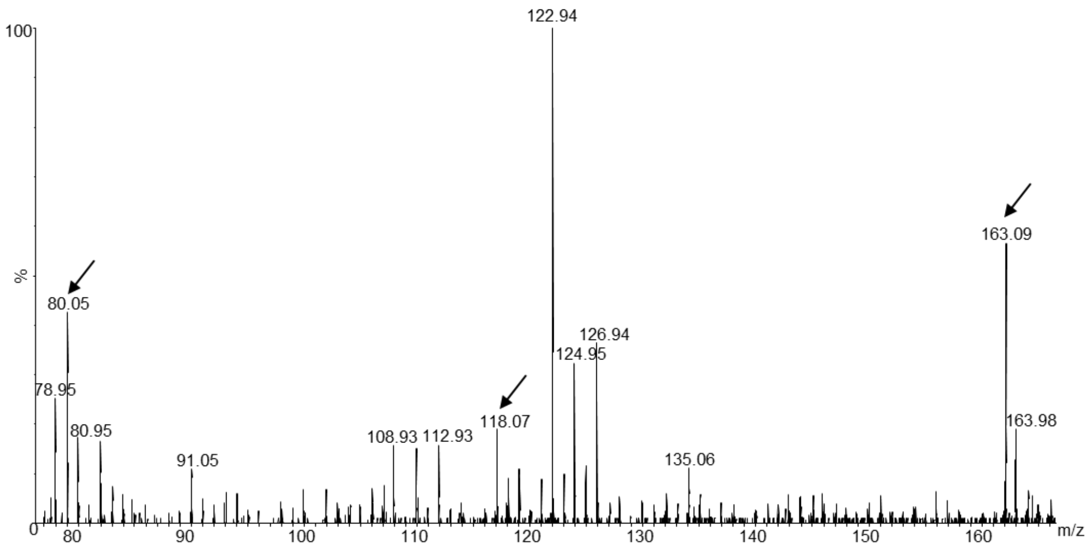

Figure S9. Structural validation of *m/z* 163.087 with CE-MS/MS.

The MS/MS spectrum of norcotinine was obtained using a transfer collision energy ramp of 10-60 eV at 0.5 Hz and a precursor isolation width of ±0.015 Da. Prominent peaks at m/z 80.05 and 118.07 correspond to the published multiple reaction monitoring transitions commonly employed for the quantification of norcotinine using triple quadrupole MS/MS analysis.

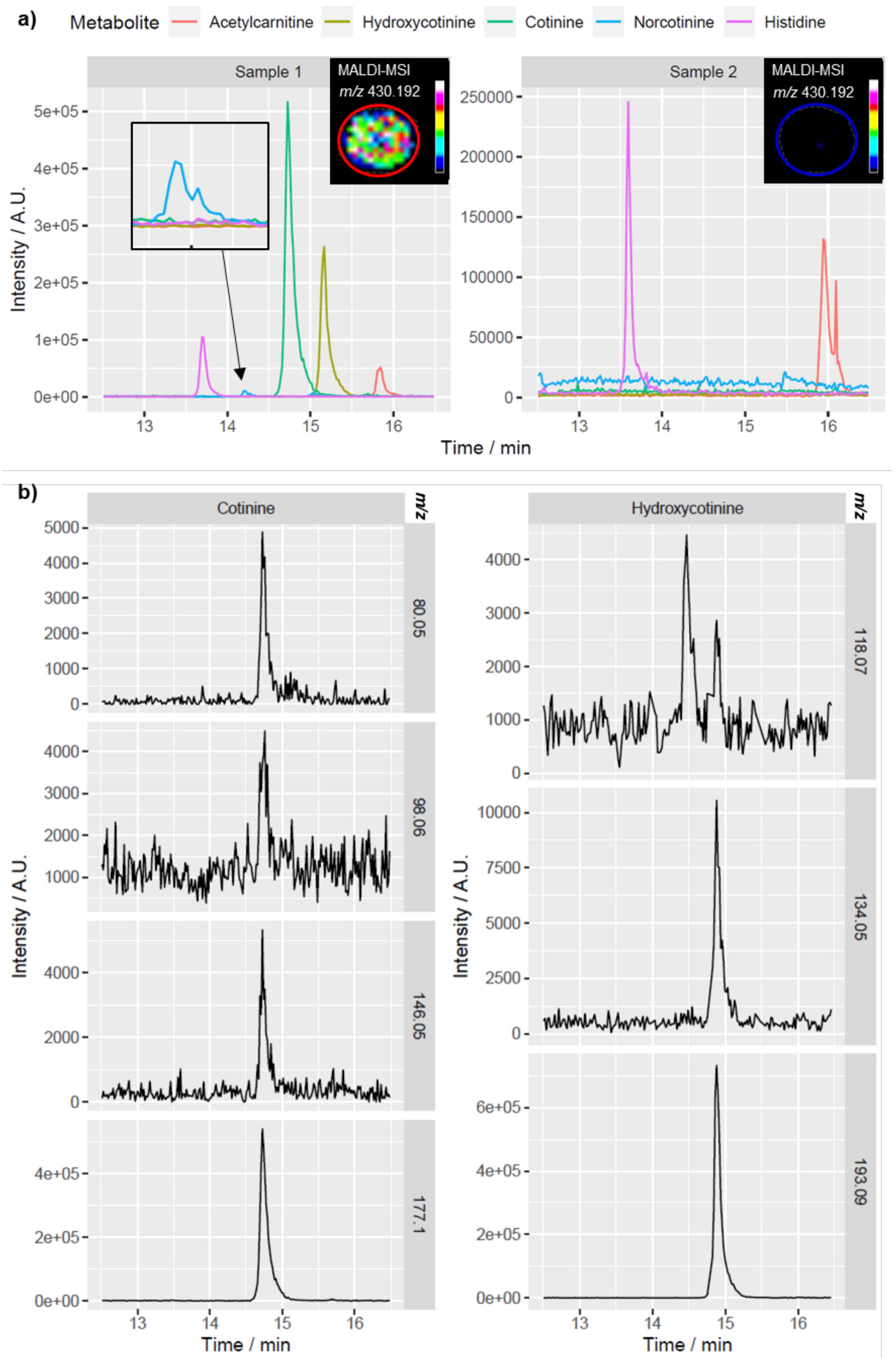
a)
Metabolite
Acetylcarnitine
Hydroxycotinine
Cotinine
Norcotinine
Histidine
Sample 1
Sample 2
MALDI-MSI
m/z 430.192
Intensity / A.U.
Time / min
b)
Cotinine
Hydroxycotinine
m/z
80.05
98.06
146.05
177.1
118.07
134.05
193.09

Figure S10. Extracted ion electropherograms from CE–MS separations.

(a) Extracted ion electropherograms (EIEs) obtained from CE–MS separations of two crude CSF samples. MALDI-MSI revealed the presence of the *m/z* 430.192 (ion image scaled to 50 % of max intensity) ion in sample 1, and subsequent analysis by CE–MS confirmed the presence of peaks corresponding to norcotinine, cotinine, and hydroxycotinine. The migration order and relative abundances observed under the experimental conditions were consistent with the reported levels of these metabolites in biofluids. Histidine and acetylcarnitine were utilized as reference compounds. When the *m/z* 430.192 ion was not detected in CSF using MALDI-MSI analysis, no putative nicotine metabolites were observed upon analysis by CE–MS. **(b)** EIEs reveal previously reported transitions of cotinine and hydroxynorcotinine resulting from in source decay of the parent ions during CE–MS analysis of crude CSF samples. Differences in migration time between panels (a) and (b) can be attributed to variances in separation potentials employed for the respective analyses. Step size 200 x 200 µm, laser size ~80 µm, 180 pixels/spot, runtime ~3 min/spot, mass resolution 220,000 at *m/z* 450.

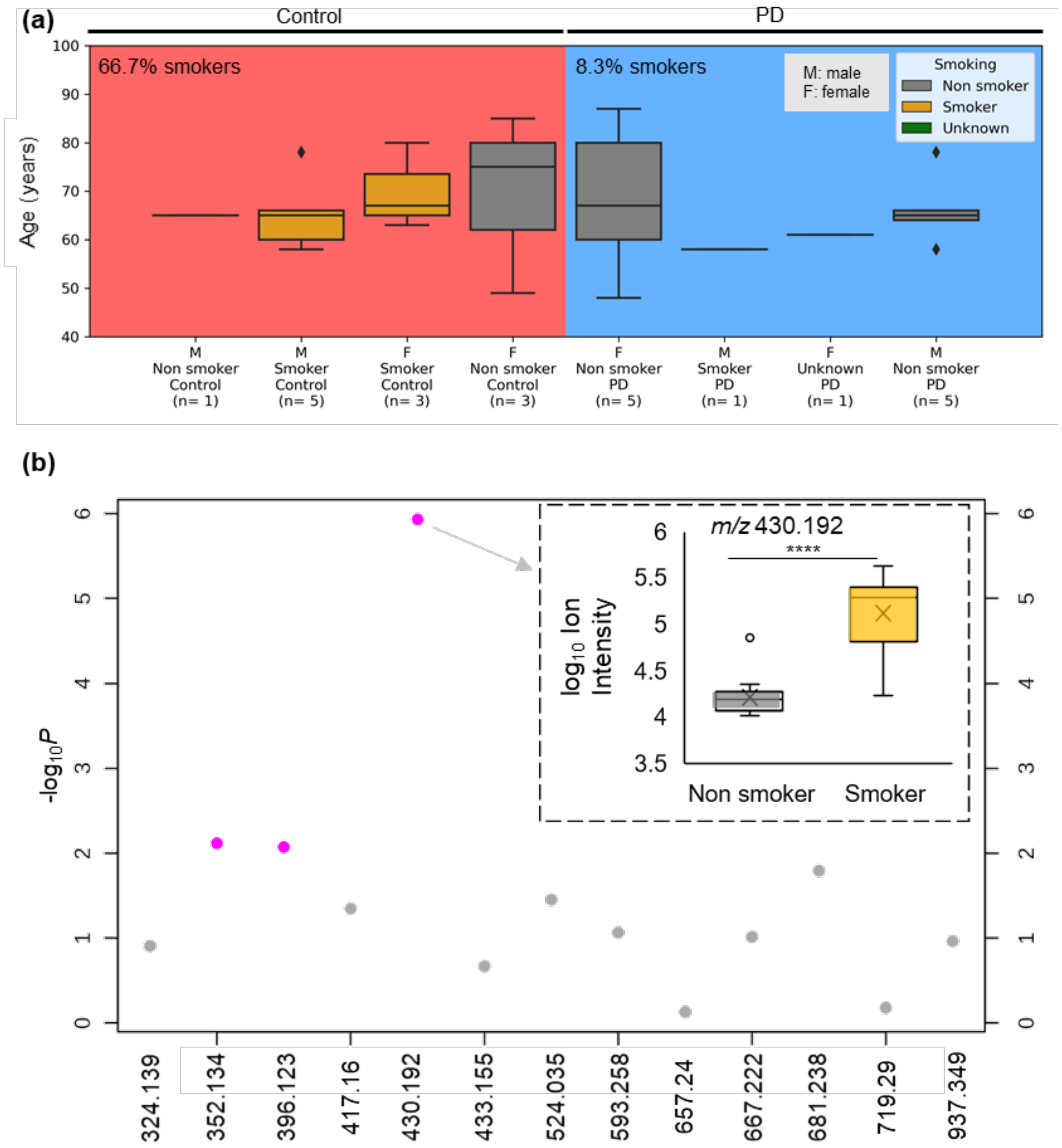

Figure S11. Effect of tobacco use on the investigated CSF metabolites.

(a) Distribution of tobacco users among control subjects and *de novo* Parkinson's disease (PD) patients based on age (y-axis) and gender (male/female). **(b)** Two-tailed *t*-tests (FDR < 0.05) were conducted to assess the impact of tobacco use on specific *m/z* values (x-axis) that exhibited significant differences between control and de novo PD samples. The y-axis represents the negative logarithm of the raw p-value, with magenta highlighting the m/z values that reached FDR < 0.05.

Supporting Tables

Table S1. Limit of detection (LOD) of selected deuterated neurotransmitters and metabolites.

**Compound**	**LOD (ng/µl)**	**Endogenous levels of corresponding NTs in blank CSF (ng/µl)**	**Equation**	**R^2**	**Linear range (ng/µl)**
HVA-d_5 †	0,025	0.11	y = 2E+06x + 30798	R^2 = 0.9989	0.013-1.1
GABA-H2O-d_6 †	0,034	0.23	y = 2E+06x + 70724	R^2 = 0.9989	0.04-1.1
5HT-d_4 †	0,024	0.01*	y = 774916x + 12164	R^2 = 0.999	0.013-1.1
3-MT-d_4†	0,08	ND	y = 536001x + 37267	R^2 = 0.9982	0.013-3.2
NE-d_6‡	0,05	0.15	y = 404836x + 66690	R^2 = 0.9909	0.013-3.2
DA-d_4 ‡	0,016	ND	y = 1E+06x + 30047	R^2 = 0.9989	0.04-0.4
L-DOPA ‡	0,38	ND	y = 130213x + 40776	R^2 = 0.998	0.35-9.5

† Molecule + 1FMP-10
‡ Molecule + 2FMP-10
* peak present but below LOD

The *m/z* peak corresponding to GABA-d_6 after the loss of water was preferably used for detection of isotope labeled GABA-d_6 because it was detected with better sensitivity.

Table S2. Mass accuracy of isotope-labeled standards and corresponding endogenous NTs obtained from the LOD experiment.

Deuterated compounds	Derivatization species	Theoretical *m/z*	Experimental *m/z*	Error (ppm)
GABA-H_2O-d_6	$[M + FMP\text{-}10]^+$	359.20250	359.203	1.39
GABA-d_6	$[M + FMP\text{-}10]^+$	377.21307	377.2134	0.89
5-HT-d_4	$[M + FMP\text{-}10]^+$	448.23215	448.2324	0.57
5-HT-d_4	$[M + 2FMP\text{-}10]^+$	701.32130	701.3213	0.01
HVA-d_5	$[M + FMP\text{-}10]^+$	455.20137	455.2015	0.29
DA-d_4	$[M + FMP\text{-}10]^+$	425.21616	425.2165	0.79
DA-d_4	$[M + 2FMP\text{-}10]^+$	678.30531	678.3049	-0.60
L-DOPA-d_3	$[M + FMP\text{-}10]^+$	468.19971	468.2002	1.04
L-DOPA-d_3	$[M + 2FMP\text{-}10]^+$	721.28886	721.2892	0.47
NE-d_6	$[M + FMP\text{-}10]^+$	443.22363	443.2242	1.29
NE-d_6	$[M + 2FMP\text{-}10]^+$	696.31278	696.312	-1.12
3-MT-d_4	$[M + FMP\text{-}10]^+$	439.23181	439.2322	0.88
3-MT-d_4	$[M + 2FMP\text{-}10]^+$	692.32096	692.3203	-0.95

Endogenous compounds	Derivatization species	Theoretical *m/z*	Experimental *m/z*	Error (ppm)
DA	$[M + FMP\text{-}10]^+$	421.19106	ND	
DA	$[M + 2FMP\text{-}10]^+$	674.28020	ND	
3-MT	$[M + FMP\text{-}10]^+$	435.20671	435.2074	1.60
5-HT	$[M + FMP\text{-}10]^+$	444.20704	444.2074	0.81
HVA	$[M + FMP\text{-}10]^+$	450.16999	450.1704	0.92
L-DOPA	$[M + 2FMP\text{-}10]^+$	718.270032	ND	
NE	$[M + 2FMP\text{-}10]^+$	690.27512	ND	
GABA-H_2O	$[M + FMP\text{-}10]^+$	353.16484	353.1652	1.02
GABA	$[M + FMP\text{-}10]^+$	371.17541	371.1758	1.06

Table S3. List of synthetic standards used for structure validation of CSF detected metabolites and the theoretical *m*/*z* values of all potential derivatization species. Only feasible derivatization species are included.

Compound	M+FMP-10	M+2[FMP-10]-CH_3 $^+$	M+2[FMP-10]-H^+	M+3[FMP-10]-2CH_3 $^+$	M+3[FMP-10]-CH_3 + -H^+	M+3[FMP-10]-2H+
3,4-DHPA	434.1751	687.2642	701.2799			
DHMPE	479.1965	732.2857	746.3013	985.3748	999.3905	1013.4060
3-MT	435.2067	688.2959	702.3115			
3-OMD	479.1965	732.2857	746.3013			
4-Hydroxy-benzaldehyde	390.1489	643.2380	657.2537			
5,6-Dihydroxyindole[a]	417.1598	670.2489	684.2646	923.3381	937.3537	951.3694
5-HIAA	459.1703	712.2595	726.2751			
5-HT	444.2070	697.2962	711.3118	950.3853	964.401	978.4166
Acetyl-histamine	421.2023	674.2914	688.3071			
Adenine	403.1666	656.2557	670.2714			
Adenosine[b]	535.2088					
Alanine	357.1598					
Arginine	442.2238	695.3129	709.3286	948.4021	962.4177	976.4334
Aspartate	401.1496					
Benseraside	525.2127	778.3018	792.3175	1031.391	1045.407	1059.422
Carbidopa	494.2074	747.2966	761.3122	1000.386	1014.401	1028.417
Creatine	399.1816	652.2707	666.2864			
Creatinine	381.1710	634.2601	648.2758			
Cystein	389.1318					
Dopamine	421.1911	674.2802	688.2959	927.3694	941.385	955.4007
Dimethoxy-phenethylamine	449.2224					
DOPAC	436.1543	689.2435	703.2591			
DOPEG	438.1700	691.2591	705.2748			
Epinephrine	451.2016	704.2908	718.3064	957.3799	971.3956	985.4112
GABA[c]	371.1754					
Glutamic acid	415.1652					
Glutamine	414.1812					
Glycine	343.1441					
Histamine	379.1917	632.2809	646.2965			
Histidine	423.1816	676.2707	690.2864			
Hordenine	433.2274					
HVA	450.1700					
Hypoxantine	404.1506	657.2397	671.2554			
Isoleucine	399.2067					
Kynurenine[d]	476.1969					
L-Carnosine	494.2187	747.3078	761.3235			
L-DOPA	465.1809	718.2700	732.2857	971.3592	985.3748	999.3905
Leucine	399.2067					
Leukoa.chr.	419.1754	672.2646	686.2802	925.3537	939.3694	953.385
L-Methyltyrosine	463.2016	716.2908	730.3064			

Lysine	414.2176	667.3068	681.3224			
Methionine	417.1631					
Norepinephrine	437.1860	690.2751	704.2908	943.3643	957.3799	971.3956
Normetanephrine	451.2016	704.2908	718.3064			
Norsalsolinol	433.1911	686.2802	700.2959	939.3694	953.385	967.4007
Octopamine	421.1911	674.2802	688.2959			
Phenylalanine	433.1911					
Proline	383.1754					
Putrescine	356.2121	609.3013	623.3169			
SAH	652.2337					
Salsolinol	447.2067	700.2959	714.3115	953.385	967.4007	981.4163
SAM[e]	667.2566					
Serine	373.1547					
Spermidine	413.2700	666.3591	680.3748	919.4483	933.4639	947.4796
Spermine	470.3278	723.4170	737.4326	976.5061	990.5218	1004.537
Taurine	393.1267					
Threonine[e]	387.1703					
α-Tocopherol	698.4932					
Tryptamine	428.2121	681.3013	695.3169			
Tryptophane	472.2020	725.2911	739.3068			
Tyramine	405.1961	658.2853	672.3009			
Tyrosine	449.1860	702.2751	716.2908			
Valine	385.1911					
Vanillactic Acid	480.1805					
VMA	466.1649					
Acetaminophen	419.1754					
5-Hydroxy-kynurenamine	448.2020	701.2911	715.3068			
4-hydroxyphenyl-acetic acid	420.1594					

Abbreviations: 3,4-dihydroxyphenylacetone, 3,4-DHPA; 3,4-dihydroxyphenylalanine methyl ester, DHMPE; 3-methoxytyramine, 3-MT; 3-methoxy-L-tyrosine, 3-OMD; 5-hydroxyindole-3-acetic acid, 5-HIAA; 5-hydroxytryptamine, 5-HT; 3,4-dihydroxyphenylacetic acid, DOPAC; 3,4-dihydroxyphenyl glycol, DOPEG; gamma-aminobutyric acid, GABA; homovanillic acid, HVA; L-3,4-dihydroxy-phenylalanine, L-DOPA; leukaminochrome/5,6-dihydroxyindoline, leukoa.chr.; S-adenosyl homosysteine, SAH; S-adenosyl L-methionine, SAM; 4-hydroxy-3-methoxymandelic acid, VMA.

[a] Could not be dissolved/not detected
[b] Detected as adenosine-H_2O, *m/z* 517.1985
[c] Also detected as GABA-H_2O, *m/z* 353.16484
[d] Detected as Kynurenine-COOH, *m/z* 430.19167
[e] Not detectable

Table S4. Univariate statistical validation (two-tailed *t*-test) of the inspected high VIP *m/z* values.

m/z	***t*-stat**	***P***	**FDR**
417.16023	-2.9796	0.0069	0.0929
937.34857	-2.9306	0.0077	0.0929
433.15514	-2.6507	0.0146	0.0984
430.19191	2.3822	0.0263	0.0984
719.2895	2.3640	0.0273	0.0984
681.23809	-2.2952	0.0316	0.0984
524.03499	-2.2434	0.0353	0.0984
667.22241	-2.2158	0.0374	0.0984
324.13869	-2.1732	0.0408	0.0984
352.13367	-2.1370	0.0440	0.0984
396.12309	-2.1248	0.0451	0.0984
593.25803	-2.0060	0.0573	0.1146
657.23974	-1.7583	0.0926	0.1710

Table S5. Untargeted univariate statistical analysis (two-tailed *t*-test) of the tobacco use effect.

m/z	*t-stat*	*P*	*m/z*	*t-stat*	*P*
***430.192**	-6.863	8.77E-07	400.211	2.3068	0.031351
489.149	-4.1881	0.000414	471.171	2.3	0.031805
327.096	-4.0824	0.000534	668.270	2.2837	0.032902
422.223	-3.9047	0.000816	*524.035	2.2834	0.032923
*396.124	3.1568	0.004756	663.312	2.2828	0.032964
*352.134	3.0177	0.006551	399.035	2.2786	0.033258
398.041	-3.014	0.006606	376.170	2.275	0.033507
635.233	2.8563	0.009454	754.346	2.2737	0.033597
853.323	-2.8171	0.010326	368.129	2.2618	0.034439
*681.238	2.8159	0.010355	1046.425	-2.2601	0.034566
679.222	2.8131	0.010418	507.194	-2.2517	0.035171
513.239	2.7971	0.0108	370.144	2.248	0.035442
561.205	-2.6063	0.016488	428.150	2.2427	0.035839
669.273	2.5449	0.018851	556.193	-2.2189	0.037644
543.167	-2.5104	0.020318	313.142	2.2183	0.037693
441.182	2.4185	0.024752	*417.160	2.2097	0.038366
555.178	-2.3977	0.025873	917.217	-2.1945	0.039588
423.207	2.3955	0.025995	793.284	-2.1871	0.040195
703.285	2.3826	0.026717	555.168	-2.1636	0.042174
384.160	2.3736	0.027236	699.094	2.1538	0.043025
302.100	-2.36	0.028028	820.012	-2.1481	0.043525
1044.299	-2.3536	0.028413	988.281	2.1182	0.046256
819.356	-2.3479	0.028754	399.143	2.1053	0.047474
559.182	-2.3326	0.029697	593.259	2.0925	0.048716
695.255	2.3186	0.030585	499.223	2.0856	0.049406
440.234	2.307	0.031338	551.292	-2.0814	0.049824

* *m/z* values found significantly altered between *de novo* PD patients and control
